# Retraction: The inhibition of tumor protein p53 by microrna-151a-3p induced cell proliferation, migration and invasion in nasopharyngeal carcinoma

**DOI:** 10.1042/BSR-2019-1357_RET

**Published:** 2021-10-29

**Keywords:** Apoptosis, Luciferase reporter assay, Matrix metalloproteinases

This article is being retracted from *Bioscience Reports* at the request of the Editor-in-Chief and the Editorial Board following receipt of a notification from a reader, alerting the Editorial Board to the inhibitor panel in Figure 3C, which contains a significant number of data points overlapping with that from figures in other publications by different authors.

The authors have been contacted in regards to the Retraction, and have not responded to the Journal's queries and the concerns raised. Given the extent of the issues raised, the Editorial Board stand by the decision to retract the article.

